# The role of ERK1/2 signaling in diabetes: pathogenic and therapeutic implications

**DOI:** 10.3389/fphar.2025.1600251

**Published:** 2025-05-09

**Authors:** Hanlin Xu, Tao Liu, Yanfen Dai, Na Li, Zhanqi Cao

**Affiliations:** ^1^ Department of Thoracic Surgery, The Affiliated Hospital of Qingdao University, Qingdao, China; ^2^ Department of Pharmacy, The Affiliated Hospital of Qingdao University, Qingdao, China; ^3^ Department of Hyperbaric Oxygen, The Affiliated Hospital of Qingdao University, Qingdao, China

**Keywords:** ERK1/2, MAPK, diabetes, complications, inhibitors

## Abstract

ERK1/2 (extracellular signal-regulated kinase 1/2) is an important member of the MAPK (mitogen-activated protein kinase) family and is widely involved in many biological processes such as cell proliferation, differentiation, apoptosis and migration. After activation by phosphorylation, ERK1/2 can be transferred into the nucleus and directly or indirectly affect the activity of transcription factors, thereby regulating gene expression. More and more studies have shown that ERK1/2 plays an important role in diabetes and its complications, such as insulin secretion, islet β cell function, diabetic cardiomyopathy, diabetic nephropathy, renal fibrosis, lipogenesis, diabetic vasculopathy, etc. These effects reveal the complexity and diversity of the ERK1/2 signaling pathway in the pathogenesis of diabetes, and its activation and inhibition mechanisms in multiple physiological and pathological processes provide potential targets for diabetes treatment. The purpose of this mini-review is to explore the key role of ERK1/2 in diabetes and the progress of research on targeted inhibitors of ERK1/2, which provides new strategies for the treatment of diabetes.

## 1 Introduction

Diabetes is a chronic metabolic disorder, whose main characteristic is hyperglycemia, that is, abnormally elevated glucose levels in the blood. Diabetes is one of the most common and fastest-growing diseases in the world, and is expected to affect 693 million adults by 2045 (Cho et al., 2018). The occurrence of this disease is related to insufficient insulin secretion or impaired insulin function. Insulin is a hormone secreted by beta cells in the pancreas, responsible for transferring blood glucose into cells for energy use (American Diabetes Association, 2011). Type 1 diabetes is usually caused by a complete loss of function of islet beta cells, and patients rely on insulin injections to control blood glucose. Type 2 diabetes, on the other hand, is associated with insulin resistance and a relative lack of insulin secretion, and is more common in adults, especially obese people. The complications of diabetes are very serious, and long-term high blood sugar can cause damage to organs such as the eyes, kidneys, nerves, heart and blood vessels, and even function failure (Cole and Florez, 2020). In addition, people with diabetes have a significantly increased risk of cardiovascular disease, one of the leading causes of death. Although there are various treatments for diabetes, there is currently no complete cure. The management of diabetes complications is also a major challenge, and current treatments, which are mostly symptomatic, do not address these issues at their root. Therefore, it is necessary to find new therapeutic targets or methods for the treatment of diabetes.

Mitogen-Activated Protein Kinase (MAPK) signaling pathway is an important intracellular signaling pathway, which is widely involved in the regulation of various physiological and pathological processes of cells, including cell proliferation, differentiation, stress response, inflammatory response and β-cell dedifferentiation (Guo et al., 2020). Members of the eukaryotic MAPK family mainly include p38, extracellular signal-regulated kinase (ERK) and c-Jun N-terminal kinase (JNK) (Saleem, 2024). ERK protein family is an important member of MAPK family, which is mainly involved in many physiological processes such as cell growth, differentiation, survival and apoptosis. The ERK family includes several subfamilies, of which ERK1 and ERK2 are the most widely studied members, and they play key roles in cell proliferation, differentiation, metabolism, and transcriptional regulation (Lu and Malemud, 2019). ERK1/2 is activated by the Ras-Raf-MEK-ERK signal transduction cascade. Phosphorylated ERK1/2 can then enter the nucleus and affect gene expression, which is closely related to cell growth, proliferation, differentiation, migration and survival (Mutlak and Kehat, 2015). It was found that the ERK1/2 signaling pathway plays a key role in diabetes and its complications, and its activation is associated with a variety of pathological processes (Ye et al., 2012; Xu et al., 2016; Ajay et al., 2024). Thus, by inhibiting the ERK1/2 signaling pathway, diabetic tissue damage and complications may be effectively alleviated, which provides new ideas and targets for future treatment.

## 2 ERK1/2 and progression of diabetes

### 2.1 ERK1/2 and insulin resistance

Insulin resistance (IR) refers to the body’s weakened response to insulin signals, resulting in the inability of insulin to effectively promote glucose uptake and utilization, resulting in high blood glucose and increased insulin secretion. Abnormal insulin signaling is one of the core mechanisms leading to IR. The insulin signaling pathway is primarily initiated by the insulin receptor, which subsequently activates a series of signaling molecules such as insulin receptor substrates (IRS) and protein kinase B (Akt). Insulin binds to its specific receptor, triggering receptor dimerization and activation of IRS-1/2, which in turn recruits and activates phosphatidylinositol 3-kinase (PI3K). PI3K can regulate glycogen synthesis, lipogenesis and glucose uptake by activating downstream Akt protein, which further activates mammalian target of rapamycin (mTOR) and glycogen synthase kinase 3 β (GSK3β) and other key proteins (Le et al., 2023). Under IR conditions, the PI3K/Akt signaling pathway is often inhibited, resulting in a decrease in the phosphorylation level of Akt protein, which leads to a reduction in the expression of glucose transporter 4 (GLUT4) and a decline in glucose uptake capacity (Roberts et al., 2013). In addition to the main PI3K/Akt signaling pathway, abnormalities in other insulin signaling pathways, such as MAPK, AMP-activated protein kinase (AMPK) and Janus kinase/signal transducer and activator of transcription (JAK/STAT) signaling pathway, etc., can also directly or indirectly cause IR. IR runs through the whole process of the occurrence and development of type 2 diabetes, and is closely related to many complications (Taylor, 2013). The role of ERK1/2 signaling pathway in IR is mainly reflected in its inhibition of insulin signaling and reduction of metabolic effects. The ERK1/2 signaling pathway is activated in a variety of cell types, and its overactivation is strongly associated with IR (Yao et al., 2022). As is known, phosphorylation of ERK1/2 is negatively regulated by dual-specificity phosphatase 6 (DUSP6). DUSP6 is a bispecific phosphatase that specifically dephosphorylates ERK1/2, thereby inhibiting its activity. Dusp6-knockout mice showed increased fat mass and decreased insulin sensitivity on a high-fat diet (Pfuhlmann et al., 2017).

**FIGURE 1:**
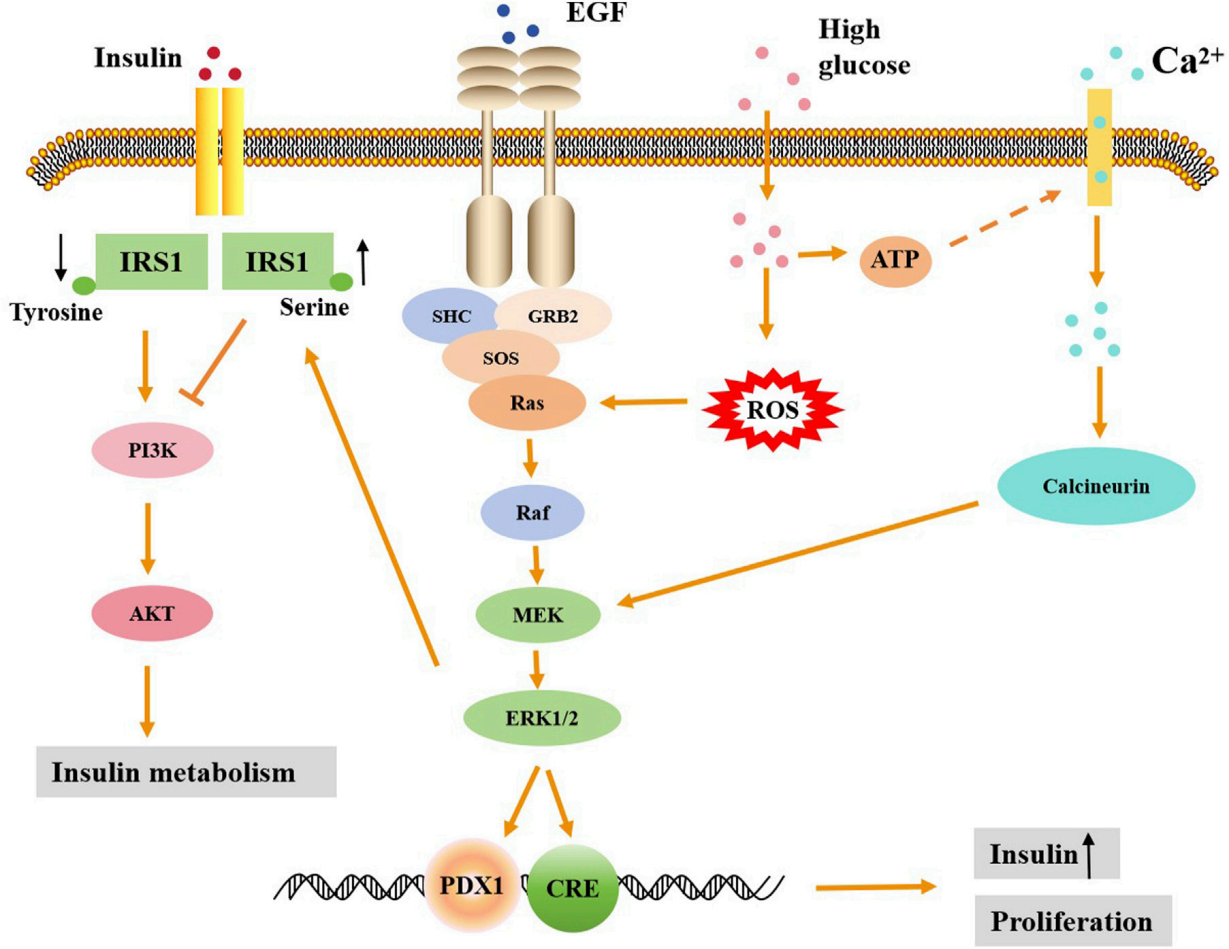
The regulatory mechanism of ERK1/2 signaling in promoting insulin secretion and insulin resistance. High glucose can activate the ERK1/2 signaling pathway by increasing Ca^2+^ influx and ROS production, leading to the entry of activated ERK into the nucleus. ERK1/2 in the nucleus can then activate downstream promoters, promoting the proliferation of islet β cells and the secretion of insulin. Activated ERK1/2 can lead to increased serine phosphorylation and decreased tyrosine phosphorylation of RS1, thereby inhibiting the downstream PI3K/Akt signaling pathway of insulin and ultimately causing insulin metabolism disorders.

Persistent ERK1/2 activation in the liver of thin mice leads to liver glycogen accumulation, fasting hyperglycemia and IR (Jiao et al., 2013). In obese mouse models, ERK1/2 knockouts significantly improved systemic insulin and glucose tolerance, suggesting that by reducing ERK activity, IR could be alleviated and metabolic health improved (Jiao et al., 2013). Studies have shown that insulin can independently activate ERK1/2 through the cAMP/protein kinase A (PKA) pathway, promoting its phosphorylation even under non-physiological conditions (Dalle et al., 2004). However, when glucose concentrations are elevated, overactivation of ERK1/2 interferes with insulin signaling, leading to a decrease in the response of the insulin gene promoter, which induces IR (Nandipati et al., 2017). For example, in patients with non-alcoholic fatty liver disease (NAFLD) and diabetes, hepatocyte-derived fibrinogen-related protein 1 (HFREP1) induces IR by activating the ERK1/2 signaling pathway, inducing hepatocyte proliferation and disrupting insulin signaling in peripheral tissues (Wu H.-T. et al., 2016). In obese mice and human adipose tissue, activation of ERK1/2 can induce insulin receptor substrates 1 (IRS1) serine phosphorylation, thereby inhibiting IRS1 tyrosine phosphorylation (Tanti and Jager, 2009; Li X. et al., 2014). Serine phosphorylation then reduced the interaction between IRS1 and PI3K and inhibited the association between IRS1 and insulin receptors (Tanti and Jager, 2009). Under normal circumstances, insulin promotes the transfer of the glucose transporter 4 (GLUT4) from storage vesicles to the cell membrane by activating the IRS and PI3K signaling pathway, thereby increasing glucose uptake and maintaining blood glucose balance (Jaldin-Fincati et al., 2017). However, in the state of IR, PI3K/Akt is not effectively activated, which inhibits the translocation of the GLUT4, thereby reducing glucose uptake and metabolism (Figure 1). Moreover, some studies have suggested that overactivation of ERK1/2 may promote IR through other mechanisms. For example, it can interfere with insulin signaling by promoting the expression of inflammatory factors such as monocyte chemoattractant protein-1 (MCP-1) (Wu H.-T. et al., 2016), or affect blood vessel function by inhibiting the sensitivity of endothelial cells to insulin (Li X. et al., 2014). The production of insulin-induced cytokines in macrophages is also involved in the induction of IR in hepatocytes through ERK1/2 and κB kinase β (IKKβ) activation inhibitors. This indicates that the activation of these two kinases leads to the inhibition of serine phosphorylation in insulin receptor substrates (IRS), thereby blocking the action of insulin (Manowsky et al., 2016). Therefore, these results indicate that the mechanism by which ERK1/2 leads to insulin resistance is complex.

### 2.2 ERK1/2 and islet β cell function

Islet β cells are important endocrine cells in the pancreas, which are mainly responsible for secreting insulin to regulate blood glucose level. ERK1/2 plays an important role in glucose-stimulated insulin secretion. High glucose stimulation leads to increased Ca^2+^ inflow in islet β cells to activate ERK1/2, which in turn regulates insulin gene expression and insulin secretion (Longuet et al., 2005; Yu et al., 2011; Martin-Vega and Cobb, 2023). The activation of ERK1/2 can promote the ability of islet β cells to sense glucose and enhance insulin secretion by regulating calcium channels (such as calcineurin) (Duan and Cobb, 2010; Osborne et al., 2012; Martin-Vega and Cobb, 2023). ERK1/2 regulates transcription of the insulin gene by phosphorylating specific transcription factors, such as PDX1 and BETA2 (Lawrence et al., 2005). This mechanism is particularly evident under glucose stimulation, suggesting that ERK1/2 plays an important role in the regulation of insulin gene expression (Figure 1). In the context of diabetes, a hyperglycemic environment overactivates the ERK1/2 signaling pathway, leading to dysfunction of islet β cells. For example, prolonged exposure to high blood glucose leads to inhibition of insulin gene transcription, and this inhibition depends on the overactivation of ERK1/2 (Lawrence et al., 2008; Luo et al., 2014). Although ERK1/2 is generally thought to promote insulin secretion in response to glucose stimulation, it has also been shown that inhibiting ERK1/2 activity does not necessarily reduce insulin secretion. For example, the use of the MEK inhibitor PD98059 inhibited the activation of ERK1/2 but did not significantly reduce insulin secretion (Wauson et al., 2013). This may be because the role of ERK1/2 signaling pathway in insulin secretion is not single or direct, but the result of interaction with other signaling pathways. In addition, insulin secretion is also affected by other factors, such as Ca^2+^ signaling, mitochondrial function, and intracellular metabolic status. Insulin secretion is a complex multi-step process involving multiple signaling pathways and molecular mechanisms. For example, insulin secretion is not only dependent on the activation of ERK1/2, but may also involve other signaling pathways such as JNK, p38 MAPK, NF-κB, etc (Nandipati et al., 2017; Chen et al., 2023). Therefore, even when ERK1/2 is activated, it may fail to significantly affect insulin secretion due to compensatory action or competitive inhibition of other signaling pathways.

ERK1/2 plays an important role in islet β cell proliferation and survival. It was found that the activation of ERK1/2 could protect β cells from apoptosis while enhance their ability to adapt to a high-glucose environment (Panse et al., 2015). MEK1/2 deficient mice with ERK signaling missing in β cells showed insufficient insulin production and reduced β cell proliferation (Ikushima et al., 2021). Some pharmacological agents such as cloverleaf factor 2, genistein, and urea oxide induce proliferation of mouse β cells *in vitro* and *in vivo* by activating ERK1/2, which can be eliminated by ERK1/2 inhibition (Fu et al., 2010; López-Acosta et al., 2013; Orime et al., 2013). Importantly, one study showed that the level of phosphorylated ERK1/2 was significantly reduced in islets of patients with type 2 diabetes (Ikushima et al., 2021), thus demonstrating the promotion of ERK1/2 phosphorylation on islet cell proliferation. However, there is a different view that in the state of hyperglycemia, the overactivation of ERK1/2 may lead to the dysfunction of islet β cells, including impaired insulin secretion and apoptosis (Yeo et al., 2012). Insulin secretagogues such as GLP-1 (glucagon-like peptide-1) inhibit the overactivation of ERK1/2, thereby protecting islet β cells from damage (Panse et al., 2015). Moreover, in a hyperglycemic environment, inhibition of ERK1/2 activity can reduce the apoptosis and dysfunction of β cells (Fu et al., 2010). This data suggests that in the context of diabetes, overactivation of ERK1/2 may lead to dysfunction and increased apoptosis islet β cell.

## 3 ERK1/2 and diabetic complications

### 3.1 ERK1/2 and diabetic cardiomyopathy

**FIGURE 2:**
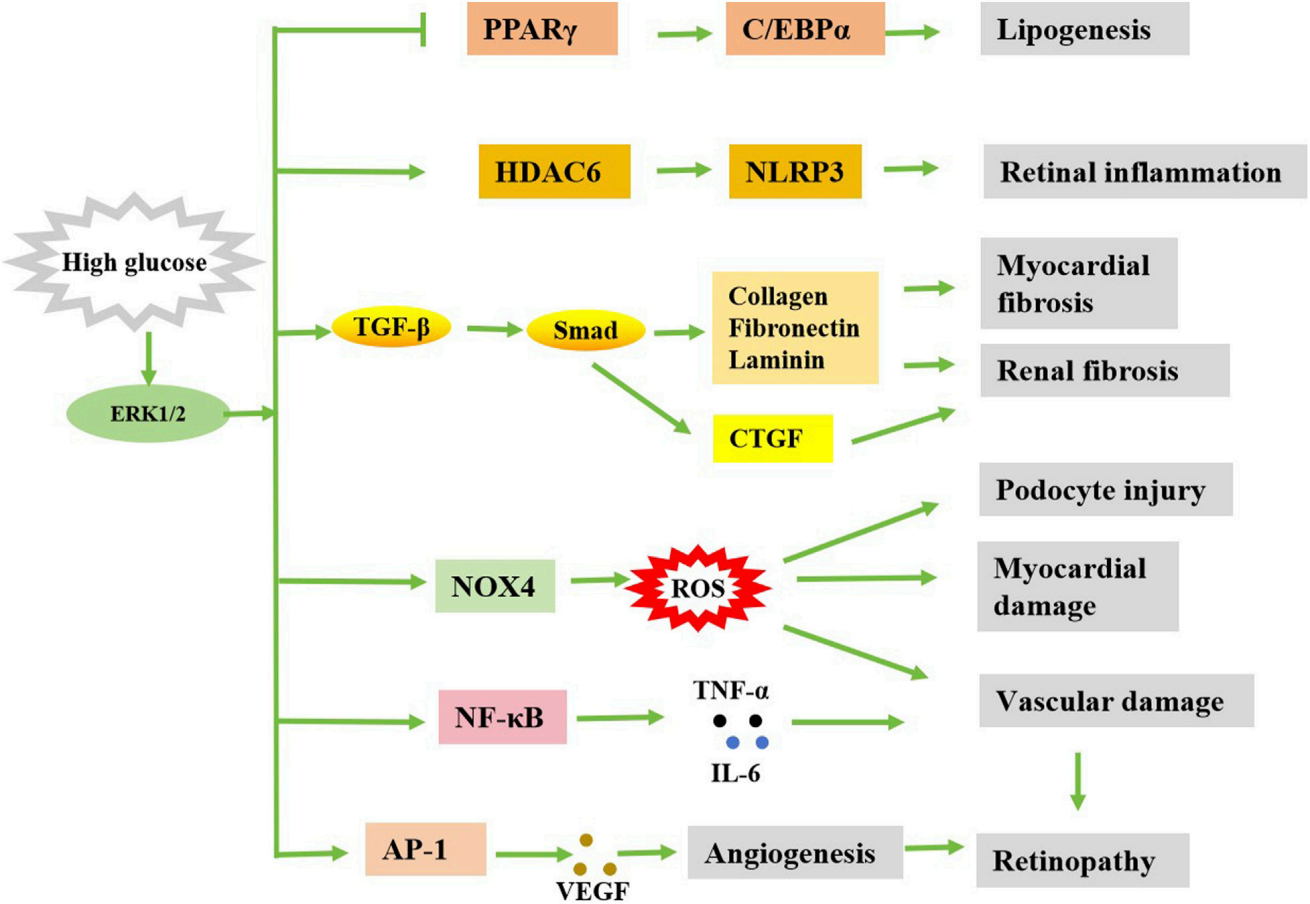
Simple diagram of the possible regulation of diabetic complications by ERK1/2. Stimulated by high glucose, activated ERK1/2 can cause diabetes complications in a variety of ways.

Diabetic cardiomyopathy (DCM) is a type of cardiomyopathy that occurs in the state of diabetes, that is, extensive focal necrosis of the myocardium on the basis of microvascular disease, and leads to subclinical cardiac dysfunction, and eventually progresses to arrhythmia, heart failure and cardiogenic shock, etc. Studies have shown that ERK1/2 plays a role in regulating myocardial oxidative stress, apoptosis, myocardial hypertrophy and myocardial fibrosis, and these processes are related to DCM (Xu et al., 2016). Dong et al. established a model of hyperglycemic cardiomyocyte hypertrophy and found that high glucose increased the volume of cardiomyocytes and the expression of hypertrophic genes, and upregulated the expression of ERK1/2, but did not increase the activity of p38 MAPK and JNK, the other two subfamilies of MAPK (Dong et al., 2017). This suggests that the activation of ERK1/2 can promote the proliferation and growth of cardiomyocytes, leading to myocardial hypertrophy, which may eventually further burden the heart. It was showed that myocardial hypertrophy was improved by drug inhibition of MEK-ERK1/2 signaling pathway (Zhang et al., 2023). In addition, reactive oxygen species (ROS) play an important role in the occurrence and development of DCM. An early study showed that antioxidant therapy can inhibit the increase of reactive oxygen species levels in cardiomyocytes and block the activation of ERK1/2, thereby preventing the resulting heart enlargement (Tanaka et al., 2001). A later study showed that the ERK1/2 inhibitor U0126 also alleviated hyperglycemic cardiomyocyte damage by increasing cell viability, reducing the number of apoptotic cells and reactive oxygen species production (Sager et al., 2016). These results suggest that inhibition of the ERK1/2 pathway can inhibit the production of ROS, thereby alleviating the progression of DCM (Figure 2).

Many studies suggest that ERK1/2 activation plays a deleterious pro-apoptotic role in inflammation, oxidative stress, remodeling, and apoptosis of the diabetic heart. For example, overactivation of ERK1/2 may lead to increased apoptosis of cardiomyocytes and deterioration of cardiac function (Huang et al., 2016; Ni et al., 2016). Therefore, the researchers used different drugs to inhibit ERK1/2 activation in the heart and cardiomyocytes under high glucose conditions, ultimately achieving the purpose of preventing DCM. Ni et al. utilized mito-TEMPO to inhibit the development of DCM using a db/db transgenic type 2 diabetes (T2DM) and streptozotocin (STZ) -induced type 1 diabetes (T1DM) model (Ni et al., 2016). mito-TEMPO is one of the superoxide dismutase (SOD) mimics that target mitochondrial ROS. Their findings suggest that the therapeutic inhibition of mito-TEMPO on mitochondrial ROS reduces adverse cardiac changes and alleviates myocardial dysfunction in diabetic mice. In addition, the study showed that the protective effect of mito-TEMPO is related to downregulation of ERK1/2 phosphorylation. Inhibition of ERK1/2 prevents high glucose from stimulating cell death in cardiomyocytes. However, other studies have shown that ERK1/2 has anti-apoptotic effects, suggesting that ERK1/2 may also have a potential protective mechanism against DCM (Yao et al., 2012; Wang et al., 2014). Activation of ERK1/2 has been reported to protect the heart during ischemia/drug induced deficiency/reperfusion (Gross et al., 2007; Lambert et al., 2014). ERK1/2 inhibitor U0126 blocks the protective effects of hydroxychloroquine in the heart during ischemia/reperfusion injury (Bourke et al., 2015). This effect of ERK1/2 may be related to different disease models. Under diabetic conditions, ERK1/2 is continuously and excessively activated, leading to deterioration of cardiac function. However, during acute myocardial injury, it plays a role in protecting myocardial cells. Therefore, intervention strategies for DCM targeting ERK1/2 signaling pathways need to be adjusted according to specific pathological conditions.

Myocardial fibrosis is another important sign of cardiac dysfunction in DCM. Transforming growth factor β (TGF-β) can promote the release of collagen from fibroblasts and cause myocardial fibrosis. A study showed that the expression of TGF-β and p-ERK1/2 is upregulated under high glucose conditions, and this expression of TGF-β is downregulated by U0126, suggesting that ERK1/2 is involved in the expression of TGF-β induced by high glucose (Wu H. et al., 2016). Zhao et al. (Zhao et al., 2024) demonstrated that N-acetylglucosaminyltransferase V (GnT-V) knockdown could reduce integrin β1 expression, inhibit downstream ERK1/2 activation, disrupt TGF-β1/Smads signaling pathway, and alleviate myocardial fibrosis induced by high glucose, accompanied by decreased expression of ANP, BNP and β-MHC. This indicates that high glucose can increase the activity of ERK1/2 in cardiac fibroblasts, and ERK1/2 inhibitors PD98059 or U0126 can inhibit the proliferation and collagen expression of high-glucose induced fibroblasts (Wu H. et al., 2016). In a recent study, U0126 was found to inhibit ERK1/2 activation in streptozotocin-induced diabetic mice, leading to a significant reduction in the expression of genes associated with hypertrophy and fibrosis. Mechanistically, ERK1/2 activation enhances the expression of fatty acid metabolism genes in the heart, such as PPARα, CPT1A and FACS, and is reversed by U0126 treatment (McLean et al., 2025). These studies suggest that blocking ERK1/2 signaling may have a protective effect on DCM.

### 3.2 ERK1/2 and diabetic retinopathy

Diabetic retinopathy (DR) is one of the common chronic complications of diabetes. It is a series of eye diseases caused by retinal vascular damage caused by long-term hyperglycemia. DR affects nearly 40% of people with diabetes, it is one of the leading causes of blindness in people under 50, with an incidence of about 4.8% (Solomon et al., 2017; Deswal et al., 2020). One of the main features of DR is the formation of new blood vessels, and the ERK1/2 signaling pathway plays a key role in this process. ERK1/2 signaling pathway could promote the secretion of vascular endothelial growth factor (VEGF), which induces angiogenesis. Studies have shown that diabetic induced retinal oxidative stress can activate the ERK1/2 signaling pathway, thereby increasing the expression of VEGF in Muller cells (Ye et al., 2010; 2012). The ERK1/2 signaling pathway was activated rapidly 1 week after diabetes induction. The downstream transcription factor AP-1 of ERK1/2 is also activated, and VEGF is highly regulated in a similar trend, which can be inhibited by the ERK1/2 inhibitor U0126 (Ye et al., 2010). The AP-1 binding congruent sequence is contained in the promoter region of the VEGF gene and is critical for VEGF expression (Guo et al., 2022). In addition, ERK1/2 is also involved in VEGF synthesis and receptor-mediated signaling, especially under conditions of hyperglycemia (Ebrahimi et al., 2023). The activation of ERK1/2 signaling pathway not only promotes the secretion of VEGF, but also intensifies the inflammatory response and oxidative stress. It was found that the activation of ERK1/2 in the retina of diabetic mice significantly increased the expression of inflammatory factors such as interleukin-6 (IL-6), tumor necrosis factor-α (TNF-α), and matrix metalloproteinase-9 (MMP-9) (Mohammad et al., 2013). These inflammatory factors can further aggravate the development of DR. ERK1/2 signaling pathway also plays a key role in the process of apoptosis and autophagy of retinal cells. Studies have shown that the activation of ERK1/2 in diabetic retina promotes the expression of histone deacetylases 6 (HDAC6), thereby increasing the level of apoptosis and autophagy, and these pathological changes can be mitigated by inhibiting the ERK1/2 signaling pathway (Cai et al., 2017). The increased expression of HDAC6 could also mediate NLR-family-pyrin-domain-containing-3 (NLRP3) inflammasome activation in diabetic conditions, leading to retinal inflammation and degeneration (Kim et al., 2025). GLP-1 therapy significantly reduces retinal cell apoptosis by inhibiting the ERK1/2-HDAC6 signaling pathway, thereby improving DR symptoms (Cai et al., 2017). In addition, ERK1/2 is involved in other signaling pathways associated with neovascularization, such as the upregulation of cyclooxygenase-2 (COX-2) and prostaglandin E2 (PGE2) (Li T. et al., 2014), which in turn affect changes in retinal blood vessels (Zhou et al., 2024). Although the ERK1/2 signaling pathway is generally considered a pro-disease factor in DR, some studies also point to a possible neuroprotective effect in the early stages. For example, it has been shown that in the early stages of diabetic retinopathy, the activation of ERK1/2 can reduce the neurotoxicity induced by high glucose and protect retinal neurons (Moustardas et al., 2023). The activation of ERK1/2 may reduce the damage of retinal cells caused by oxidative stress by inducing the expression of antioxidant enzymes such as transcription factor NF-E2-related factor 2 (Nrf2) (Ren et al., 2016). This may in some cases be related to the anti-apoptotic effect of ERK1/2. It may also be related to the degree of activation of ERK1/2 or the pathological stage of DR. The pro-apoptotic or anti-apoptotic effects of ERK1/2 may depend on the environment and cell type in which it is activated. In conclusion, the role of ERK1/2 in DR is mainly reflected in promoting the release of VEGF, aggravating inflammatory response and oxidative stress, affecting apoptosis and autophagy, and participating in neovascularization (Figure 2).

### 3.3 ERK1/2 and diabetic vasculopathy

Diabetic vasculopathy refers to the damage of the systemic vascular system caused by long-term hyperglycemia, including the lesions of large blood vessels and microvessels (Jia et al., 2024). The occurrence of these lesions is closely related to many factors such as hyperglycemia, insulin resistance, lipid metabolism disorder, inflammatory reaction and oxidative stress. The role of ERK1/2 in diabetic vasculopathy is mainly reflected in the activation and regulation of its signaling pathway, which plays an important role in diabetic vascular dysfunction and pathological process. In the hyperglycemic environment, the activated ERK1/2 signaling pathway promotes the migration and proliferation of vascular smooth muscle cell (VSMC), thus participating in the occurrence of diabetic vasculopathy. For example, it was showed that activation of ERK1/2 significantly increased the proliferative potential of VSMC in the aorta of rats under conditions of hyperglycemia (25 mmol/L) (He et al., 2012). In addition, ERK1/2 activation could affect the cell cycle by inhibiting the expression of related genes (such as p21, p27), thus promoting the proliferation of VSMC (Wei et al., 2022; Koval et al., 2023). ERK1/2 signaling pathway not only participates in the proliferation of VSMC, but also significantly affects its migration ability. It was found that Ang II-induced ERK1/2 activation promoted VSMC migration (Yoshizumi et al., 2020). The activation of ERK1/2 signaling pathway is closely related to VSMC migration under hyperglycemia conditions (Shi et al., 2015; Shi et al., 2015017). This excessive proliferation and migration of VSMC will further aggravate diabetic vascular disease (Huang et al., 2024). In the hyperglycemic environment, the activation of ERK1/2 signaling pathway is closely related to oxidative stress. For example, in a diabetic rat model, the activation of ERK1/2 increases the expression of non-phagocytic nicotinamide adenine dinucleotide phosphate (NADPH) oxidases 4 (NOX4), a major source of ROS in the vasculature, which in turn causes oxidative stress and exacerbates vascular damage (Rozentsvit et al., 2017). Moreover, activation of ERK1/2 promotes the release of inflammatory factors, such as interleukin (IL-6), tumor necrosis factor (TNF-α), etc., which further aggravate diabetic vasculopathy (Ebrahimi et al., 2023). Some researchers have confirmed that after the phosphorylation level of ERK1/2 is reduced, the calcification of endothelial cells can be significantly reduced (Zhan et al., 2015), which suggests that vascular calcification may be related to the activation of ERK1/2 pathway. Although the ERK1/2 signaling pathway plays multiple roles in diabetic vasculopathy, the detailed mechanism by which ERK1/2 affects diabetic vasculopathy is still not fully understood.

### 3.4 ERK1/2 and diabetic nephropathy

Diabetic nephropathy (DN) is one of the serious complications of diabetes, and it is also the main cause of end-stage renal disease. 30%–40% of patients with diabetes will eventually develop end-stage renal disease (Tang and Yiu, 2020). According to Sakai et al. 's findings, p-ERK1/2 is expressed in kidney mesangial cells, endothelial cells, podocytes, renal tubular epithelial cells and renal interstitial infiltrated monocytes to varying degrees in both normal and diabetic patients (Sakai et al., 2005). In diabetic patients, the number of p-ERK1/2 positive cells in the glomeruli is correlated with the degree of glomerular damage, suggesting that the phosphorylation level of ERK1/2 plays a certain role in the process of glomerular injury of human DN. Previous studies have shown that hyperglycemic stimulation can activate ERK1/2 in renal tubule cells, leading to cell hypertrophy and increased extracellular matrix synthesis (Fujita et al., 2004; Zheng et al., 2025). Similarly, under the stimulation of hyperglycemia, renal tubular epithelial cells developed hypertrophy and increased the expression of TGF-β, while the application of ERK1/2 pathway inhibitor PD98059 could significantly inhibit the hyperglucose-induced hyperexpression of TGF-β and extracellular matrix (ECM) in mesangial cells and tubular epithelial cells (Xu and Kyriakis, 2003). Increased expression of TGF-β in DN activates the synthesis mechanism of ECM (such as collagen, fibronectin, lamectin, etc.), and participates in the process of diabetic glomerular hypertrophy and progressive accumulation of extracellular matrix, thus leading to glomerular sclerosis. In a high glucose environment, the ERK1/2 signaling pathway is activated, promoting the expression of TGF-β1 and other factors, thereby accelerating the epithelial-to-mesenchymal transition (EMT) in renal tubular cells, leading to renal interstitial fibrosis (Zha et al., 2019). ERK1/2 can also promote the expression of connective tissue growth factor (CTGF) by activating TGF-β, which in turn promotes renal fibrosis (Wang et al., 2009a; Wang et al., 2009b). CTGF is an indicator of fibrosis and a cytokine that promotes renal fibrosis in the occurrence and development of DN (Toda et al., 2018). The upregulation of TGF-β1 induced CTGF was attenuated by inhibiting the activation of ERK1/2 (Cheng et al., 2015). Moreover, the increase of TGF-β can further activate ERK1/2, promote the synthesis of ECM, accelerate glomerular sclerosis and renal interstitial fibrosis (Hayashida et al., 2003). Therefore, ERK1/2 and TGF-β may have mutually active effects and synergistically promote the development of DN (Figure 2).

In addition, ERK1/2 can aggravate kidney injury by promoting inflammatory response, affecting oxidative stress, and regulating podocyte function. For example, it was found that ERK1/2 can further promote kidney injury by regulating the expression of inflammatory factors such as interlukin-6 (IL-6) and tumor necrotic factor-α (TNF-α) (Liu et al., 2017). Activation of ERK1/2 can lead to increased production of ROS, which in turn triggers oxidative stress, leading to cell dysfunction and death. Overproduction of ROS in the kidney can activate a variety of intracellular signaling pathways that initiate the expression and transcription of genes responsible for cell proliferation, ultimately leading to the deposition of excess extracellular matrix and the formation of the DN (Singh et al., 2011). As is known, podocytes are important cell types for maintaining the integrity of the glomerular filtration barrier. While the ERK1/2 could further aggravate podocyte injury and glomerulosclerosis by inhibiting podocyte proliferation and promoting its apoptosis and migration (Chen et al., 2022). Moreover, phosphorylation of ERK1/2 is negatively regulated by DUSP6 in DN. For example, in a model of DN, DUSP6 expression is decreased in hyperglycemic environments, and DUSP6 overexpression can reduce the apoptosis and inflammatory response of podocyte induced by high glucose, suggesting that DUSP6 protects cell function by inhibiting the ERK1/2 signaling pathway (Chen et al., 2020). Upregulation of DUSP6 alleviates renal fibrosis, while its absence leads to decreased glucose tolerance (Wang et al., 2023). These data indicate that ERK1/2 is involved in the pathogenesis of DN mainly by promoting cell transdifferentiation, inflammatory response and apoptosis.

### 3.5 ERK1/2 and lipogenesis and metabolic diseases

Lipogenesis and metabolism disorders are common complications of diabetes. This leads to high levels of lipids in the blood, which thickens, hardens and thickens the lining of blood vessels, increasing the risk of cardiovascular disease. ERK1/2 is one of the important signaling pathways that drive the proliferation and differentiation of adipocytes (Figure 2). Studies have shown that the activation of ERK1/2 is essential for the early differentiation of fat cells, but its overactivation can lead to obesity and inflammation of adipose tissue (Kassouf and Sumara, 2020). For example, in obese mouse models, the loss of ERK1/2 reduces inflammation in adipose tissue and liver steatosis, thereby protecting the mice from IR (Jager et al., 2011). In addition, ERK1/2 promotes lipogenesis by promoting the activity of PPARγ (peroxisome proliferator-activated receptor γ) and maintaining the stability of lipogenic transcription factors (Than et al., 2013). However, excessive ERK1/2 signaling inhibits the activity of lipogenesis related transcription factors such as PPARγ and C/EBPα, leading to inhibition of lipogenesis (Liao et al., 2008; Gwon et al., 2013; Zang et al., 2013). For example, yams significantly inhibit adipocyte differentiation by inhibiting the phosphorylation of ERK1/2 (Gwon et al., 2013). These data suggest that ERK1/2 has a dual role in adipogenesis: moderate activation promotes adipogenesis, while excessive activation may inhibit adipogenesis. In addition to its role in lipogenesis, ERK1/2 also plays a key role in the process of lipolysis. It promotes the release of fatty acids by regulating the α-adrenaline and cAMP-dependent pathways and activation of the β3-adrenergic receptor (β3AR) (Robidoux et al., 2006; Kassouf and Sumara, 2020). This effect helps to maintain energy balance, but overactivation can lead to hyperlipolysis, which can lead to metabolic disorders.

## 4 Inhibitors against ERK1/2 pathway in diabetes treatment

To date, many researchers have made efforts to develop ideal ERK1/2 pathway inhibitors for the treatment of diabetes. Currently marketed inhibitors targeting the MAPK pathway mainly inhibit the direct upstream kinase MEK of ERK1/2, and inhibition of MEK can indirectly inhibit the activity of ERK1/2, such as trametinib and selumetinib, which have shown good efficacy in the treatment of tumors. Although there are few research data on the application of ERK1/2 inhibitors in diabetes, and they are mainly concentrated in the animal experimental stage or even *in vitro* experiments, their research in the field of tumor therapy provides a new idea for the treatment of diabetes. The effects of several direct or indirect ERK1/2 pathway inhibitors on diabetes are shown in Table 1.

**TABLE 1:** Several direct or indirect ERK1/2 pathway inhibitors.

Inhibitor	Target	Model	Outcomes	Ref
U0126	MEK/ERK	STZ-induced diabetic mice	Inhibited cardiac fatty acid metabolism alleviated diabetic cardiomyopathy	McLean et al. (2025)
PD98059	MEK/ERK	Cerebral ischemia-reperfusion injury model in diabetic rats	Reduced nerve damage and relieve symptoms	Liu et al. (2022)
Geniposide	P2Y14/ERK	BMECs model of cerebral ischemia *in vitro*	Inhibited the release of proinflammatory cytokines IL-8, MCP-1 and IL-1β	Li et al. (2016)
FR180204	ERK1/2	WT and Pak-1-KO mice treated with isoproterenol	Decreased ERK1/2 phosphorylation and reduced left ventricular hypertrophy	Taglieri et al. (2011)
AG1478	EGFR/ERK	Cultured VSMC treated with high glucose	Prevent vascular endothelial dysfunction caused by hyperglycemia and reduce the proliferation and migration of vascular smooth muscle cells	Akhtar et al. (2013)
FAM3D	MKP1/ERK	Mouse pancreatic αTC1-6 cells	Inhibited glucagon secretion	Cao et al. (2017)
Erianin	ERK1/2	STZ-induced diabetic mice	Reduces inflammation of the retina caused by microglia	Zhang et al. (2019)
OE-DUSP6	ERK1/2	MPC5 cells	Protects against HG-induced podocyte injury	Chen et al. (2020)

## 5 Conclusion remarks

Diabetes is a complex metabolic disease, its prevalence is on the rise, if not well controlled, will cause serious complications, seriously affect people’s health. Therefore, it is very necessary to find new therapeutic targets for diabetes. ERK1/2 is an important member of the MAPK family and is activated by the Ras-Raf-MEK-ERK signal transduction cascade. It is widely involved in cell proliferation, differentiation, survival and various physiological and pathological processes. In recent years, it has been found that overactivation of the ERK1/2 signaling pathway plays an important role in the occurrence and development of a variety of diseases, including cancer, inflammation, neurodegenerative diseases, diabetes, and cardiovascular diseases. In this mini-review, we reviewed the role of ERK1/2 in insulin tolerance, islet β cell function, and diabetic complications. Moderate ERK1/2 activation can promote insulin secretion and synthesis, while excessive activation can lead to insulin resistance and even lead to islet β cell dysfunction. In addition, overactivation of ERK1/2 may aggravate complications of diabetes, such as DCM, DR, DN, lipogenesis, and diabetic vasculopathy. Nevertheless, ERK1/2 may also play a beneficial role, such as moderate activation of ERK1/2 may promote lipogenesis, reduce neurotoxicity, and protect the heart from ischemia/drug deficiency/reperfusion. This indicates that the role of ERK1/2 signaling pathway in diabetes mellitus has a certain duality. There are two main reasons: On the one hand, the activation of ERK1/2 can promote cell proliferation, migration and adaptive response; On the other hand, overactivation may lead to apoptosis, inflammatory response and tissue damage. Some other factors, such as differences in experimental models, cell types, and pathological stages of diabetic complications, may also cause the ERK1/2 signal to play different roles. This indicates that the role of ERK1/2 in insulin secretion and diabetic complications is very complex.

Moreover, we also discussed the potential therapeutic inhibitors against ERK1/2 in the treatment of diabetes or it is complications. In specific cases, exploring inhibitors of the ERK1/2 pathway is considered a novel approach to the treatment of diabetes. Although a wide variety of inhibitors targeting the ERK1/2 signaling pathway have been reported, most of them are targeted for the treatment of tumors, and few are used in the treatment of diabetes. The inhibitors described in this review include direct inhibition of ERK1/2 phosphorylation or inhibition of ERK1/2 upstream components that exert indirect inhibition of ERK1/2 phosphorylation. Although ERK1/2 inhibitors can improve insulin gene inhibition induced by chronic hyperglycemia, some studies suggest that they may have no significant effect on insulin secretion under acute glucose stimulation (Panse., 2015; Leduc et al., 2017). This suggests that ERK1/2 inhibitors may not be able to completely replace the regulatory mechanisms of acute insulin secretion. Due to the differences in experimental conditions and research objects, its specific effects still need to be further verified and further discussed.

In conclusion, this review systematically detailed the role of ERK1/2 in the regulation of diabetes and it is complications, as well as enumerating several inhibitors of ERK1/2 signaling pathway. ERK1/2 may be a potential target for the treatment of insulin release, islet β cell dysfunction, insulin resistance and diabetic complications. Even so, the roles of ERK1/2 in the complications of diabetes are complex and varied, and may either promote disease progression or play a protective role by inhibiting inflammation and apoptosis. ERK1/2 has significant potential as a target for the treatment of diabetes and its complications, but some challenges remain. Future research needs to focus on developing more specific ERK1/2 inhibitors to reduce off-target effects, or exploring whether ERK1/2 inhibitors can be used in combination with existing diabetes drugs, which may provide new ideas for early diagnosis and precise treatment of diabetes.
